# The Extent of Consumer Product Involvement in Paediatric Injuries

**DOI:** 10.3390/ijerph13070654

**Published:** 2016-07-07

**Authors:** Jesani Catchpoole, Sue Walker, Kirsten Vallmuur

**Affiliations:** 1 Centre for Accident Research & Road Safety—Queensland, Queensland University of Technology, Kelvin Grove, Queensland 4059, Australia; k.vallmuur@qut.edu.au; 2 National Centre for Health Information Research & Training, Queensland University of Technology, Kelvin Grove, Queensland 4059, Australia; s.walker@qut.edu.au

**Keywords:** paediatric injury, injury surveillance, injury data, product-related injury

## Abstract

A challenge in utilising health sector injury data for Product Safety purposes is that clinically coded data have limited ability to inform regulators about product involvement in injury events, given data entry is bound by a predefined set of codes. Text narratives collected in emergency departments can potentially address this limitation by providing relevant product information with additional accompanying context. This study aims to identify and quantify consumer product involvement in paediatric injuries recorded in emergency department-based injury surveillance data. A total of 7743 paediatric injuries were randomly selected from Queensland Injury Surveillance Unit database and associated text narratives were manually reviewed to determine product involvement in the injury event. A Product Involvement Factor classification system was used to categorise these injury cases. Overall, 44% of all reviewed cases were associated with consumer products, with proximity factor (25%) being identified as the most common involvement of a product in an injury event. Only 6% were established as being directly due to the product. The study highlights the importance of utilising injury data to inform product safety initiatives where text narratives can be used to identify the type and involvement of products in injury cases.

## 1. Introduction

Children are more vulnerable to product-related injury and fatality compared to other age groups as they are more likely to use products in ways other than their intended use, due to their lack of understanding and cognitive ability to avoid product hazards [1,2,3]. Young children particularly, who are still in their physical and cognitive developmental stages, are at even greater risk [4]. Product safety regulators in Australia have increased their concentration on intervention for products which pose hazards to young children, and recognised the need for better use of injury data to monitor product safety issues [5]. One of the challenges in utilising injury data from hospitals for product safety purposes is that clinically coded data (using International Classification of Diseases systems) have limited ability to identify the level of consumer product involvement in an injury event [5].

Understanding the cause and how a consumer product is involved in the occurrence of an injury is integral to a comprehensive approach to preventing consumer product-related injuries [6]. One of the injury prevention issues that should be taken into account by product safety regulators is human failure, where safety instructions are often overlooked which in turn, leads to product misuse [7]. This is particularly evident among children, where ascertaining causal factors can be challenging, given products themselves are not always the main cause of injury; whereas misuse and/or other external factors are often prevalent. Understanding the product involvement in injury events also assists relevant regulators to determine the most appropriate type of intervention. Knowledge of how products are involved in the chain of an injury event can determine the degree of control and the types of intervention tools that can be introduced to prevent the injury from occurring [8].

In 2000, a categorisation system to determine product involvement was developed in a European study conducted by Bauer and Sector from the Institute Sicher Leben (Austrian Institute for Home and Leisure Safety). Called the Product Involvement Factor (PIF), it is a tool that has been used in the European injury surveillance system (formerly EHLASS) to interrogate text narrative data by using an automated keyword matching method to group injury cases into seven “involvement” categories [9].

In this study, the Product Involvement Factor (PIF) was adapted to analyse the extent of consumer product involvement in paediatric injuries (in children aged 0–17 years old) recorded in the Queensland Injury Surveillance Unit (QISU) database.

## 2. Materials and Methods

A secondary analysis of paediatric injury data was performed using QISU data collected between 1 January 2008 and 31 December 2010. QISU collects Emergency Department-based injury surveillance data based on the National Data Standards for Injury Surveillance (NDS-IS) from a convenience sample of 33 hospital emergency departments throughout Queensland, Australia. The database contains a range of data fields including the nature of injury, bodily location of injury, injury severity, intent of injury, as well as text narratives which are collected by triage nurses in emergency departments during triage assessment. All injury records sustained by children aged 0–17 years old were extracted from the database and were filtered to exclude assault, self-harm injuries and events of undetermined intent. In total, 7734 paediatric injuries were randomly selected using SPSS Statistics software (IBM Corp., Armonk, NY, USA) to represent 10% of the total paediatric injuries recorded in the QISU database.

The selected sample cases were reviewed manually by the lead author to determine any product involvement in the injury event. The text narrative data were used as the main source of information to classify the 7734 paediatric injury cases into the eight categories of Product Involvement Factors (PIF) Table 1:

In order to assist the classification process, a set of directive questions adapted from Bauer and Sector’s study were used as a guide in assigning the most appropriate product involvement category. One of the challenges in assigning PIF categories is that the inclusions for each category can overlap to some degree. As a result, the hierarchy of questions in assigning category 3, 4, 5 and 6 was modified slightly by reversing the order of questions to avoid more cases classified under the proximity category. Instead of placing the most inclusive category (PIF 3–Proximity) in the beginning of the hierarchy, the order was reversed placing the most exclusive category (PIF 6—High intrinsic risk) at the beginning of the hierarchy. Furthermore, to adjust to the Australian product safety setting, an eighth category was added to separate the products regulated by the Australian product safety regulators from those under the responsibility of other regulators. For example—motor vehicles, food, medications and building structures are regulated separately in Australia by other regulatory organisations.

Following the PIF assignment, a blind coding exercise was conducted on the same set of data, and a Kappa statistic was used to test the reliability of the coding.

## 3. Results

In total, 44% of the 7734 reviewed cases were associated with consumer products in which different types of involvements were identified. As displayed in Table 2, the most common involvement of a product in an injury was due to a proximity factor, with 25% of reviewed cases being categorised under this group. This comprised more than half (56%) of all product-related injuries. The type of product involvement could not be established due to inadequate descriptions of how the injury occurred for 13% of cases.

In total, consumer products played a significant role in the occurrence of the injury in just over 6% of all reviewed cases. This included approximately 4% of cases associated with high intrinsic risk products, 2% of injury cases due to products being maladapted or misused and under 1% caused by defective products.

Approximately 19% of the injuries reported in the reviewed sample were associated with non-manufactured products and 22% had insufficient information to identify whether an object was involved or not. Approximately 15% of injuries reviewed were under other regulations and, as a result, the product involvement among these cases was not further classified.

Further analysis to examine how the categorisation of product involvement applied in different age groups was conducted. The analysis demonstrated that younger children were more likely to be involved in consumer product-related injuries. As displayed in Table 2, proximity-related incidents comprised the highest proportion of injuries in children aged 0–9 years old. In contrast, older children were more likely to be injured in non-consumer product incidents with almost 30% of injuries among children aged 13–15 years associated with non-manufactured products. Approximately 27% of injuries amongst 10–12 year olds and 24% amongst 16–17 year olds had no record of consumer-product involvement in the injury.

The reliability of the PIF classification was tested using a Kappa Statistical analysis after blind coding by a second coder was conducted. The result shows a substantial level of agreement between the two coders, K = 0.641 (95% CI, 0.628 to 0.655), *p* < 0.0005. Of the approximately 27% of cases coded differently by the second coder, 70% of the disagreement were cases coded under the proximity category. Differences were resolved by re-emphasising the hierarchy of directive questions used in assigning PIF categories, where proximity as the most inclusive category was placed at the bottom of the hierarchy. Thereafter, an additional blind coding phase was conducted for cases where the two coders previous had conflicting categorisations, resulting in almost complete agreement (K = 0.96 (95% CI, 0.954 to 0.965)).

### 3.1. Injuries Related to Proximity Products

In total, 1925 injury cases were classified under the proximity-related injury category. These injuries comprised more than half (56%) of all consumer product-related injuries that were identified in the text narrative review. As shown in Table 3, proximity-related incidents were mostly sustained by younger children under the age of 10 years old with a peak in the 1–3 year old group (31%). Falls were the most dominant mechanism of injury in this category, comprising 47% of all proximity product-related injuries, with almost 70% of these injuries sustained by children under 7 years old. This was followed by injuries due to being struck by, or in contact with an object, accounting for 32%. Children aged 1–3 years old were the most prominent age group in this injury mechanism group, accounting for 27%. Furniture products were the most common product group that was associated with both falls and being struck by or in contact with an object. Fall injuries in the proximity category also included a large number of falls from playground equipment and sporting equipment.

### 3.2. Injuries Related to Defective Products

A total of five injury cases were classified under the defective product category during the text narrative review process. Two seven year old males were struck by defective products. One was hit by a broken yoyo when a ball bearing from within the object bounced into the child’s ear, while the other child was struck by a falling basketball hoop. Also included in this category, a ten year old male child fell from a swing after the support rope broke, and a six year old male was injured after the chemical release from a glow stick entered his eye following the object snapping whilst the child was playing with it. Lastly, a one year old female toddler sustained an open wound on her scalp due to falling metal blinds. The number and proportion of defective products identified in this review may be underestimated due to the large number of cases being inadequately described in the injury data.

### 3.3. Injuries Related to Maladapted or Misused Products

One hundred and sixty two cases were classified under maladaptation or misuse of products. As shown in Table 3, foreign body injuries were the most prevalent mechanism of injury in this category, comprising 54% of all maladapted or misuse related injuries. These injuries were mostly sustained by children aged 1–6 years old (84%). Coins were the most common product misused by children causing foreign body injuries. Chemical effect injuries were the second most common mechanism of injury, accounting for 24% of all injuries due to maladapted/misused products, with children aged between 1–3 years the most vulnerable age group for these injuries. The majority of injuries due to misuse of chemical products were related to household chemicals.

### 3.4. Injuries Related to High Intrinsic Risk Products

High intrinsic risk products were involved in 341 injury cases. Crushing or piercing injuries were the most common in this category, comprising 57% of all high intrinsic risk injuries. The distribution of crushing, cutting and piercing injuries in this category was relatively even in all age groups with a slight increase in children aged 10 to 12 years. Knives were the most common products causing cutting injuries, followed by other kitchen utensils. The second most common mechanism of injury under the high intrinsic risk product category was thermal effect injuries. Approximately, 26% of injuries in this category were caused by a thermal effect, with most of the injuries sustained by younger children under 4 years old (70%). Cooking appliances and hot beverages were the two most common products causing burn injuries in this category.

### 3.5. Injuries Related to Inadequate Information

In total, 977 injury cases were identified as product-related injuries; however, the injury descriptions in the data were insufficient to suggest the type of product involved. There were a significant proportion of injuries with inadequate descriptions in the data, with almost 30% of all product-related injuries included in this category. Bicycles, trampolines, scooters and skateboards were the top five products that were commonly associated with injuries under this category. Bicycle-related injuries were most common in children aged 10–15 years old, whereas trampoline-related injuries were most common in younger children aged 1–9 years old. Scooter-related injuries were most common in children aged 10–12 years whereas skateboard-related injuries were most common in children aged 13–15 years.

## 4. Discussion

The findings from the text narrative review indicate that text narrative data plays a significant role in identifying the types of products involved in injury cases, and are also necessary for investigating product involvement. The types of objects involved in the injuries were able to be retrieved from the text narrative data in almost 80% of all injury cases evaluated in the text narrative review. Types of products managed under other regulations in Australia and non-manufactured objects were also able to be separated from a range of consumer products during the PIF categorisation process. This is consistent with the findings from Jones and Lyons’s study in which text narrative data was found to reduce the proportion of unknown cases for object involved [10]. The results in this study also confirmed the findings from Bauer and Sector’s study that found that a PIF categorisation tool is useful to classify product-related cases in injury data [9].

The majority of consumer product-related injuries were related to proximity factors (25%), though 13% were inadequately described to establish the product involvement. The lack of information in the injury descriptions can possibly be explained by the commonness and regularity of several injury mechanisms and a lack of understanding of clinical staff about the need for detailed documentations of injury chains of events. For example, falls from bicycles and trampolines which comprised the highest proportion of injuries in the inadequate description category were also the most frequent injury presentations in the emergency departments and clinical staff may have considered it sufficient to document the product, but not necessarily the chain of events.

Using the text narrative review in this study has several limitations. Firstly, the results depend on the accuracy and completeness of the text data. Text narratives reviewed for this study were collected at the point of triage in the emergency department. The urgency of treatment may have contributed to the accuracy and completeness of the information in the text narrative data. Also, high intrinsic risk is a relatively volatile concept which can be interpreted differently depending on other factors (e.g., age and alcohol use). For example, one product can be perceived as having high intrinsic risk when used by a younger child but not when used by an older child. For this study, the high intrinsic risk category is limited to products that have naturally high intrinsic risks by themselves, and do not require other contributing factors for them to be qualified as such. In order to assist in the categorisation process, Haddon’s matrix tool was used as a guide to understand the transfer of energy between the factors involved in an injury event [11]. Products with thermal energy (burn) and mechanical energy (specifically sharp object) risks are examples of products classified as high intrinsic risk for the purposes of this study, as such products do not require an additional contributing factor to pose a risk to consumers.

While the results might be influenced by the proportion of unspecified cases, safety interventions can still be drawn from the results. The results show that most of the proximity-related incidents were related to falling and striking against objects and were more prominent amongst younger children. The authors identified that possible safety countermeasures can be developed through the use of protective equipment (i.e., safety barriers and helmets). Consumer products can potentially be made safer for children at the design level by eliminating hazardous features [3,5]. The results also show that majority of defective product-related incidents were related to falling objects and broken toys and play equipment. These can be addressed by enforcing proper installation and promoting more durable design for toys. Safety interventions can also be developed to address consumer product misuse-related incidents by limiting younger children’s access to chemicals and small objects to prevent them from ingesting or inserting these products. Limiting the use of small features that are easily detachable from children’s products will reduce adverse events associated with such products. Lastly, injuries caused by high intrinsic risk products were mostly related to cutting injuries especially amongst older children and burns amongst the younger children. These can be addressed by encouraging cautious use of sharp objects and heating appliances. Access to cooking areas should also be restricted for younger children. Protective barriers and use of thermal safe padding can also be recommended to prevent these injuries.

The study results also reflected differences in product involvement in different age groups where younger children were more likely to be involved in consumer product-related injuries, whereas older children were more likely to be injured in non-consumer product incidents. These findings highlight the important role of age in influencing the type of product involvement and type of injury children are prone to. The difference in the type of product involvement in different age groups could be explained by developmental stages in different age groups, with younger children likely to use products in ways other than their intended use, due to their lack of understanding and cognitive ability to avoid product hazards. Younger children are more attracted to products with bright colours and are more exposed to finished products such as toys and indoor products for children, whereas older children are more exposed to outdoor activities and sport-related equipment [2]. Instead of finished products, older children are more attracted to raw material to create new products on their own [2].

## 5. Conclusions

The study showed that utilising text narrative data plays a substantial role in identifying the types of products involved in injury cases. The study also confirmed that the Product Involvement Factor categorisation tool is useful to classify product-related cases in injury data. Although there were a significant number of unspecified cases, the information in the text narrative data can still be utilised to assist product safety regulators to understand how consumer products are involved in injury events and to determine possible safety interventions to prevent consumer product-related injuries. The study highlights the importance of utilising injury data to inform product safety initiatives, as well as the need to improve the data collection in emergency departments to better capture consumer product-related injuries and injury chains of events.

## Figures and Tables

**Table 1:** Product involvement factor categories and examples.

PIF Category	Description	Injury Narrative Example
(1) No product involved	No consumer product was mentioned in the text narrative	*“Over-exertion of lower back when running…”*
(2) Non-manufactured object/product	Injury was caused by non-manufactured object	*“Child ran and tripped over a rock…”*
(3) Proximity product	A consumer product was involved as an intermediate object in the injury event due to its physical presence	“*Child was injured when running around and fell on the edge of a coffee table…”*
(4) Defective product	A consumer product with malfunction or faulty parts was involved in the injury even	*“Swinging on hammock, hammock broke, fell off…”*
(5) Maladapted or misused product	A consumer product was purposefully used in a manner which was not its intended use or misused due to ignorance or lack of customer information on safety instruction causing injury	*“Baby capsule was placed on kitchen bench without seatbelt on, baby fell off…”*
(6) High intrinsic risk product	A product known to have inherent high risk with its use	*“Contact burn from BBQ hot plate that was just recently turned off…*”
(7) Consumer product injury with inadequate description	A consumer product was involved in the injury but there was inadequate description to inform how the injury occurred	*“Child was injured when riding a bike…*”
(8) Products regulated by other regulatory bodies	A product that is not regulated under Product Safety regulation (e.g., motor vehicles, medications, food, etc.)	*“Child was injured in a motor vehicle accident…”*

**Table 2:** Product involvement in sampled Queensland paediatric injuries 2008–2010.

	No Record of Product	Non-Manufactured Product	Proximity-Related	Defective Product	Maladapted or Misuse Product	High Intrinsic Product	Product Injuries (Inadequate Information)	Under Other Regulation
N (%)	1684 (22)	1481 (19)	1925 (25)	5 (<1)	162 (2)	341 (4)	977 (13)	1159 (15)
Mean age	8.5	9.3	6.3	6.2	3.8	7.7	4.8	5.6
Males N (%)	913 (54)	996 (68)	1107 (58)	4 (80)	96 (60)	216 (63)	679 (70)	684 (59)
**Age group (year) N (row %)**								
<1	56 (16)	42 (12)	128 (36)	0 (0)	8 (2)	23 (7)	45 (13)	54 (15)
1–3	349 (18)	249 (13)	590 (31)	1 (<1)	88 (5)	94 (5)	152 (8)	390 (20)
4–6	208 (19)	166 (15)	366 (33)	1 (<1)	42 (4)	43 (4)	153 (14)	144 (13)
7–9	244 (22)	202 (18)	299 (27)	2 (<1)	11 (1)	44 (4)	162 (15)	132 (12)
10–12	384 (27)	343 (24)	284 (20)	1 (<1)	7 (<1)	46 (3)	221 (15)	148 (10)
13–15	319 (25)	357 (28)	194 (15)	0 (0)	4 (<1)	41 (3)	193 (15)	179 (14)
16–17	124 (24)	122 (23)	64 (12)	0 (0)	2 (<1)	50 (10)	51 (10)	112 (21)

**Table 3:** Consumer product involvement in different mechanism of injury by age group.

Product Involvement by Mechanism of Injury	Age Groups N (row %)							Total N (col %)
	<1	1–3	4–6	7–9	10–12	13–15	16–17	
**Proximity product**	**128 (7)**	**590 (31)**	**366 (19)**	**299 (16)**	**284 (15)**	**194 (10)**	**64 (3)**	1925 (56) *
Fall	88 (10)	338 (37)	196 (21)	135 (15)	95 (10)	51 (6)	10 (1)	913 (47)
Struck, hit by contact with object	28 (5)	165 (27)	109 (18)	96 (16)	117 (19)	79 (13)	24 (4)	618 (32)
Acute over—exertion of body part	5 (2)	28 (13)	18 (8)	38 (18)	59 (27)	48 (22)	19 (9)	215 (11)
Crushing, cutting, piercing	5 (4)	42 (30)	34 (24)	26 (19)	11 (8)	13 (9)	8 (6)	139 (7)
Other and unspecified mechanism		7 (39)	4 (22)	1 (6)	2 (11)	1 (6)	3 (17)	18 (1)
Foreign body	1 (8)	4 (31)	4 (31)	2 (15)		2 (15)		13 (1)
Thermal effect	1 (17)	3 (50)	1 (17)	1 (17)				6 (<1)
Suffocation		2 (100)						2 (<1)
Chemical effect		1 (100)						1 (<1)
**Defective product**		**1 (20)**	**1 (20)**	**2 (40)**	**1 (20)**			5 (<1) *
Struck, hit by contact with object				2 (100)				2 (40)
Chemical effect					1 (100)			1 (20)
Crushing, cutting, piercing			1 (100)					1 (20)
Fall		1 (100)						1 (20)
**Maladapted/misuse of product**	**8 (5)**	**88 (54)**	**42 (26)**	**11 (7)**	**7 (4)**	**4 (2)**	**2 (1)**	162 (5) *
Foreign body	3 (3)	39 (44)	35 (40)	6 (7)	3 (3)	1 (1)	1 (1)	88 (54)
Chemical effect		32 (82)	2 (5)	1 (3)	2 (5)	2 (5)		39 (24)
Struck, hit by contact with object	1 (8)	7 (58)	1 (8)	2 (17)	1 (8)			12 (7)
Crushing, cutting, piercing		4 (57)	1 (14)		1 (14)		1 (14)	7 (4)
Other and unspecified mechanism	1 (17)	3 (50)	1 (17)	1 (17)				6 (4)
Suffocation	2 (33)	3 (50)	1 (17)					6 (4)
Fall	1 (33)		1 (33)			1 (33)		3 (2)
Thermal effect				1 (100)				1 (1)
**High intrinsic risk product**	**23 (7)**	**94 (28)**	**43 (13)**	**44 (13)**	**46 (13)**	**41 (12)**	**50 (15)**	341 (10) *
Crushing, cutting, piercing	5 (3)	33 (17)	29 (15)	29 (15)	35 (18)	30 (15)	34 (17)	195 (57)
Thermal effect	13 (15)	48 (55)	8 (9)	6 (7)	4 (5)	4 (5)	4 (5)	87 (26)
Struck, hit by contact with object	3 (12)	7 (28)	2 (8)	6 (24)	1 (4)	3 (12)	3 (12)	25 (7)
Foreign body	2 (11)	4 (22)	2 (11)	1 (6)	4 (22)	3 (17)	2 (11)	18 (5)
Chemical effect		2 (50)	1 (25)			1 (25)		4 (1)
Electric, radiation effect			1 (25)				3 (75)	4 (1)
Acute over—exertion of body part					1 (33)		2 (67)	3 (1)
Other and unspecified mechanism				1 (33)	1 (33)		1 (33)	3 (1)
Fall				1 (50)			1 (50)	2 (1)
**Inadequate information**	**45 (5)**	**152 (16)**	**153 (16)**	**162 (17)**	**221 (23)**	**193 (20)**	**51 (5)**	977 (29) *
Fall	35 (7)	59 (11)	81 (16)	95 (18)	132 (25)	102 (20)	17 (3)	521 (53)
Crushing, cutting, piercing	1 (1)	21 (17)	20 (16)	20 (16)	25 (20)	18 (15)	17 (14)	122 (12)
Struck, hit by contact with object	4 (3)	16 (13)	20 (17)	13 (11)	30 (25)	32 (26)	6 (5)	121 (12)
Acute over—exertion of body part	1 (1)	16 (16)	7 (7)	19 (19)	23 (23)	29 (29)	6 (6)	101 (10)
Foreign body	3 (4)	29 (41)	18 (26)	9 (13)	4 (6)	4 (6)	3 (4)	70 (7)
Other and unspecified mechanism		1 (5)	6 (30)	4 (20)	4 (20)	5 (25)		20 (2)
Chemical effect		8 (53)	1 (7)	1 (7)	2 (13)	3 (20)		15 (2)
Thermal effect		1 (20)		1 (20)	1 (20)		2 (40)	5 (1)
Suffocation	1 (50)	1 (50)						2 (<1)
**Total**	**204 (6)**	**925 (27)**	**605 (18)**	**518 (15)**	**559 (16)**	**432 (13)**	**167 (5)**	**3410 (100)**

* % of total.

## Abbreviations

The following abbreviations are used in this manuscript:

PIF	Product Involvement Factor
QISU	Queensland Injury Surveillance Unit
NDS-IS	National Data Standard for Injury Surveillance
